# Application of deep learning in the detection of breast lesions with four different breast densities

**DOI:** 10.1002/cam4.4042

**Published:** 2021-06-16

**Authors:** Hongmei Li, Jing Ye, Hao Liu, Yichuan Wang, Binbin Shi, Juan Chen, Aiping Kong, Qing Xu, Junhui Cai

**Affiliations:** ^1^ Department of Radiology Subei People's Hospital of Jiangsu Province Yangzhou Jiangsu China; ^2^ Yizhun Medical AI Beijing China; ^3^ School of Electronics Engineering and Computer Science Peking University Beijing China

**Keywords:** Yizhun AI, breast density, false‐positive, sensitivity

## Abstract

**Objective:**

This retrospective study evaluated the model from populations with different breast densities and showed the model's performance on malignancy prediction.

**Methods:**

A total of 608 mammograms were collected from Northern Jiangsu People's Hospital in Yangzhou City. The data from this province have not been used in the training or evaluation data set.

The model consists of three submodules, lesion detection (Mask‐rcnn), lesion registration between craniocaudal view and mediolateral oblique view, malignancy prediction network (ResNet). The data set used to train the model was obtained from nine institutions across six cities. For normal cases, there were no annotations. Here, we adopted the free‐response receiver operating characteristic (FROC) curve as the indicator to evaluate the detection performance of all cancers and triple‐negative breast cancer (TNBC). The FROC curves are also shown for mass/distortion/asymmetry and typical benign calcification in two kinds of populations with four types of breast density.

**Results:**

The sensitivity to mass/distortion/asymmetry for the four types of breast (A, B, C, D) are 0.94, 0.92, 0.89, and 0.72, respectively, when false positive per image is 0.25, while these values are 1.00, 0.95, 0.92, and 0.90, respectively, for the amorphous calcification lesions. The sensitivity for the cancer is 0.85 at the same false‐positive rate. The TNBC accounts for about 10%–20% of all breast cancers and is more aggressive with poor prognosis than other breast cancers. Herein, we also evaluated performance on the TNBC cases. Our results show that Yizhun AI could detect 75% TNBC lesions at the same false‐positive level mentioned above.

**Conclusion:**

The Yizhun AI model used in our work has good diagnostic efficiency for different types of breast, even for the extremely dense breast. It has a guiding role in the clinical diagnosis of breast cancer. The performance of Yizhun AI on mass/distortion/asymmetry is affected by breast density significantly compared to that on amorphous calcification.

## Graphical abstract

The performance of the Yizhun AI model is maximal as compared to the previously reported model. The performance of Yizhun AI on mass/distortion/asymmetry is affected by breast density significantly compared to that on amorphous calcification.

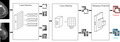

## 1 INTRODUCTION

Breast cancer is the most common cancer among malignant tumors that threaten women's health worldwide and is the second cause of cancer. Approximately 2.045 million new breast cancer cases are detected worldwide, and about 510,000 women are deceased from breast cancer in 2018.^1^ Early diagnosis and treatment improve the prognosis, prolong survival, and reduce the mortality of breast cancer patients. Because of the most cost‐effective imaging modality, mammography is one of the essential methods for breast cancer screening and the most commonly used imaging technology before surgery.^2^ The detection and identification of benign and malignant breast tumors preoperatively are important for imaging physicians. However, in the clinical study, some atypical or small lesions are often ignored or omitted because of the masking effect of dense fibroglandular tissue, often causing confusion and difficulty in diagnosis.^3^ Furthermore, several studies proved that breast density is also an independent breast cancer risk factor. According to the fifth edition Breast Imaging Reporting and Data System (BI‐RADS), breast can be categorized into four types of density^4^ (A, almost entirely fatty; B, scattered fibroglandular densities; C, heterogeneously dense; and D, extremely dense). Women with dense breasts (C or D) have a 1.5‐ to 2‐fold increased risk of breast cancer as compared to those with type B breast.^3^, ^5^ Some studies have shown that high breast density may play a critical role in tumor aggressiveness, especially in younger women.^5^ In Asians, type C and D breasts are common. The diagnosis becomes difficult with increasing density because the dense glandular and fibrous tissue may hide underlying cancer that has almost the same density as the surrounding environments.

In recent years, the number of women participating in breast cancer screening has shown a significant increase due to the application of new imaging techniques, which further increases the workload of the radiologists. Traditional imaging diagnosis relies on the subjective judgment of radiologists, who should exhibit high‐stress resistance and concentration. Long‐term and high‐load work will inevitably lead to visual and psychological fatigue. In addition, mammography is a projection imaging with certain limitations. Some small or atypical tumors are easily masked by dense glandular tissue of the breast; however, Asian women mostly have dense glands (type C and D), which are prone to be misdiagnosed and missed.^6^, ^7^, ^8^ Previous studies have shown that about 20% of the newly diagnosed breast cancer patients display abnormalities during the reexamination by mammography,^9^ indicating that the Radiology could provide false‐negative results during the previous imaging examination of these patients. In the past decades, the computer‐aided detection/diagnostic system for mammography has always been a popular research direction.^10^

Recently, deep learning as a subfield of Yizhun AI has been applied in several industries; for example, a technology convolution neural network (CNN) is a series of neural network algorithms that have made great progress in medical imaging analysis^11^, ^12^, ^13^ Compared to the traditional machine learning algorithms, neural network algorithm is based on representation learning and is more generic. It only relies on the input of raw data and allows computers to discover features that are used to build predictive statistical models automatically through a backward propagation optimization algorithm, which greatly improves the training efficiency and inference performance of the model.^14^, ^15^ A CNN deep learning model trained on a large data set of mammographic lesions shows a similar performance when used by experienced certified radiologists and outperforms a state‐of‐art traditional CAD.^16^ In order to relieve the work pressure of the doctors, a missed diagnosis is avoided, and the detection rate of breast lesions is improved. A breast artificial intelligence‐aided diagnostic system from Yizhun AI is already approved by the Chinese Food and Drug Administration. Strikingly, it has made significant progress in tumor detection in the dense breast via learning and training.

The purpose of this study is to evaluate the system about the performance of detection and diagnostic ability on breast lesions in different breast types using raw mammography data. In addition, this study would present an optimization direction for some other diseases using the Yizhun AI system described above.

## 2 MATERIALS AND METHODS

In this retrospective study, we evaluated an Yizhun AI system to detect lesions on mammograms. The mammography Yizhun AI system was substantiated in three aspects. First, we evaluated the model's performance on mass/distortion/asymmetry and amorphous calcification lesions in different breast types (A, B, C, D). Second, we assessed the performance of cancer detection in all data sets and triple‐negative breast cancer (TNBC) cases. Third, the model's prediction performance about malignancy was evaluated at the patient level.

**FIGURE 1:**
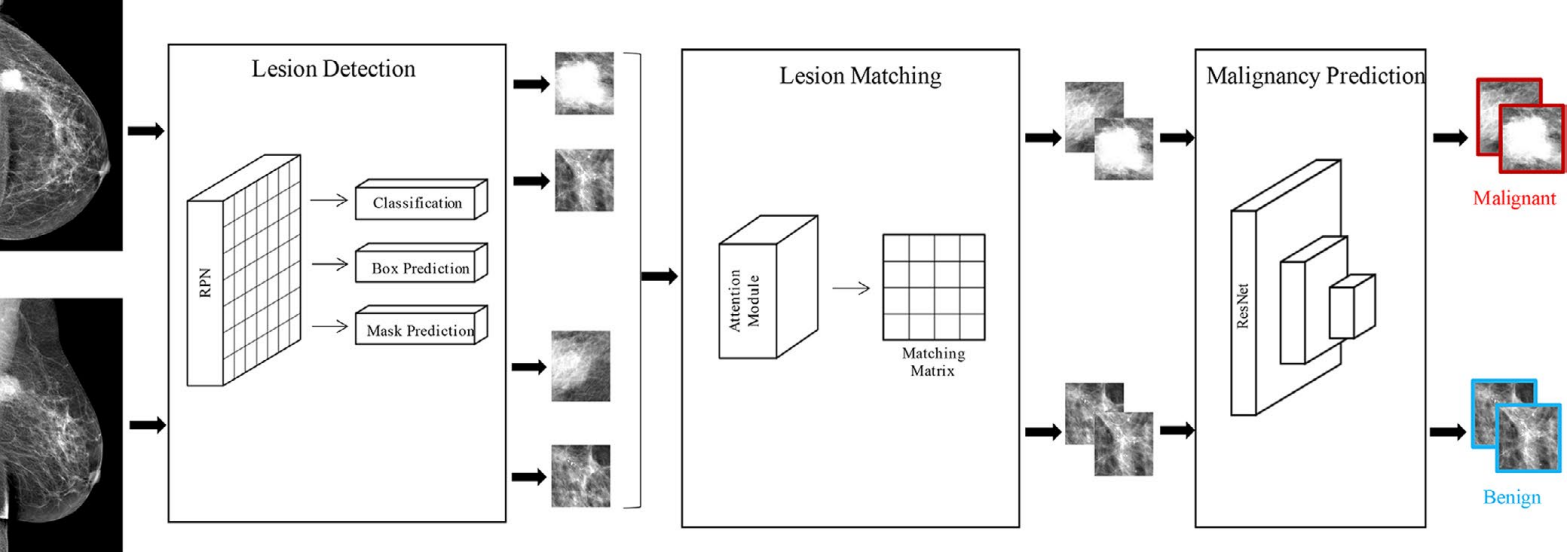
Mammography Yizhun AI autodiagnostic system architecture. There are three parts to this system. All parts consist of convolution neural networks (CNNs). The architecture and output of CNN can execute a deferent task. The first part of the system can execute three kinds of tasks, and the last two modules constitute the classification network

The mammography Yizhun AI diagnostic system mainly consists of three parts as is shown in Figure 1: lesion detection, lesion registration, and malignancy prediction modules. In the first step, the lesions were detected on craniocaudal (CC) and mediolateral oblique (MLO) views independently by a CNN‐Mask RCNN is known as the highest precision detection architecture.^17^ It is composed of two modules, Faster‐RCNN and segmentation, which have been applied to several diseases via lung nodule Yizhun AI auto diagnostics system.^18^ Since X‐Ray mammography is a two‐dimensional projection image displaying overlapping lesions. The non‐maximum suppression (NMS) algorithm is primarily used to remove the redundant candidate bounding box output from the detection model. Thus, we adopted the soft‐NMS algorithm to avoid false negatives caused by lesion overlap while reducing the redundant detection results.^19^ In the second step, the lesions from the two views were input into the matching prediction module, which will output the matching probability matrix. In the last step, the model output provides the lesion types and the BI‐RADS rating of each lesion. Next, we defined the worst lesion based on the patient's BI‐RADS score. The average inference time for each patient was <20s, depending on the number of lesions.

The data set used to train the model is collected from nine institutions across six cities of China. The density‐based distribution of the training data set is shown in Table 1. The percentage of each type of breast is the fraction of each type on the unnormal populations, while the normal cases do not have any annotations.

**TABLE 1:** Training set of breast artificial intelligence based computer‐aided detection (Yizhun AI‐CAD) system

	A	B	C	D	No lesion	Total
Train	1060	4924	20,840	2524	29,168	58,516
Percent	3.61% (1060/29356)	16.78%	71.01%	8.60%		
Validation	54	126	287	141	0	608
Percent	8.9% (54/608)	20.7%	47.2%	23.2%		

The validation data set collected from Northern Jiangsu People's Hospital in Yangzhou city consists of 608 women (438 cases were biopsied during the 1‐year follow‐up; of these, 409 cases were cancer‐positive, and 29 cases are cancer‐negative. 170 were confirmed to be benign by follow‐up for at least 1 year) and the characteristic of the validation set is shown in Table 2. Six hundred and seven women had double side breasts, and one woman had only one side breast. For each breast, both CC and MLO views were considered, and the lesions on both views were matched manually in the golden standard annotation. All the data were acquired using the GE Senographe Essential Mammography from 2015 to 2019 and were never used to train or tune any module of the system.

**TABLE 2:** Characteristics of patients in validation

	A	B	C	D
Age, year				
Mean	70	62.7	53.2	47
Interquartile range	62–77	55–70	46–59	43–51
Body thickness (mm)				
Mean	47	48	50	48
Interquartile range	39–53	41–54	53–58	40–58
Mass/distortion/asymmetry	36	96	328	174
Amorphous calcification	5	32	101	72

In this study, we adopted the free‐response receiver operating characteristic (FROC) curve to evaluate the performance of the model with respect to the detection of the lesions. FROC, introduced in the clinical problem by Chakraborty et al.,^20^ can visualize the performance of the object localization task.

All the deep learning models in the system are trained on the platform PyTorch. The ROC analysis is performed on the web‐based calculator developed by Johns Hopkins. The 95% confidence interval was obtained by the bootstrap method. The cut‐point of the model is defined by the concordance probability method which maximizes the product of sensitivity and specificity.

CZ(c)=Se(c)×Sp(c).

The statistical analyses include Chi‐Square and Kappa were performed using the SPSS software (version 26).

## 3 RESULTS

**FIGURE 2:**
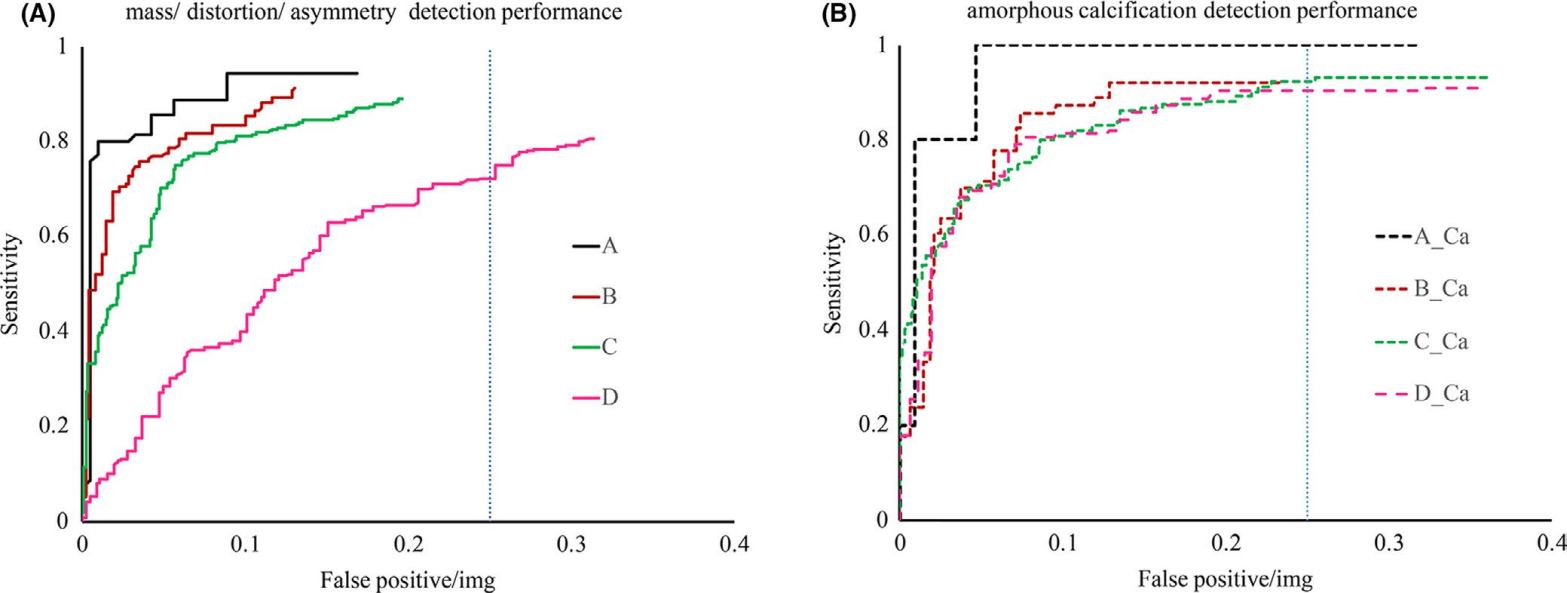
Free‐response receiver operating characteristic curves for two groups of lesions on the four types of breast. The first group includes mass, distortion, and asymmetry lesions. The second group is the amorphous calcification. The straight vertical dash line is located at the false positive rate of 0.25

For the purpose of evaluation, the detection performance of the model we plotted the FROC curve, a plot about lesion fraction (detected lesions/total number of lesions) versus non‐lesion localization fraction (false positive/total number of images) for the four types of breast. We have evaluated the performance of detection on two types of lesions (Figure 2). According to the FROC curves, mass/distortion/asymmetry lesions' curve change more than the amorphous calcification, which means mass/distortion/asymmetry is more sensitive to breast density, especially for the type of D breasts. Mammography is the golden standard for calcification, and amorphous calcification is usually related to the risk of cancer. According to the FROC curves, we can see that the model's performance on amorphous calcification is almost the same for breast types B, C, and D.

By varying the threshold for the model prediction can obtain a series of models with different sensitivity and false‐positive. We chose the threshold at which the model's false‐positive level per image is 0.25. Considering there are four views for each patient, so the average false‐positive per patient is 1. And model's sensitivity for two kinds of lesions at deferent breast types are shown on (Table 3). We also made independent Chi‐Square test for the breast type and sensitivity. For the mass/distortion/asymmetry and amorphous calcification lesions, their independent Chi‐Square test *p* value with breast types are 2.9 × 10^−7^ and 0.713 respectively.

**TABLE 3:** Sensitivity on four types of breast when false positive per image equal to 0.25

Lesion type	A	B	C	D	*p* value
Mass/distortion/asymmetry	0.94	0.92	0.89	0.72	<0.001
Amorphous calcification	1	0.95	0.93	0.90	0.713

**FIGURE 3:**
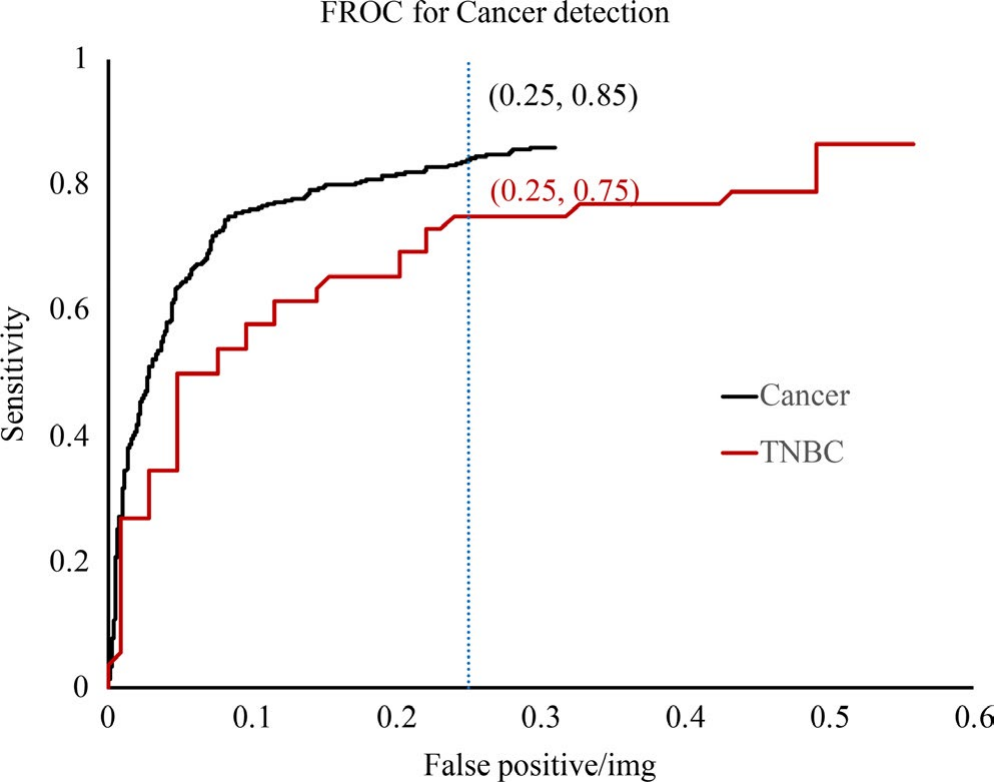
Free‐response receiver operating characteristic (FROC) curves for all cancers and triple‐negative breast cancer (TNBC). Black solid line describes the performance of whole cancer, and solid red line describes the performance of the TNBC. At the false‐positive rate of 0.25, the Yizhun AI system sensitivity for cancer and TNBC corresponds to 0.9 and 0.75, respectively

Of the 409 breast malignancies diagnosed during the study interval in the cancer cohort, 26 (6.4%) cases were TNBC which refers to the fact that the cancer tests negative for estrogen receptors, progesterone receptors, and excess HER2 protein. TNBC grows and spreads rapidly and usually with poor prognosis than other subtypes.^21^ Thus, we evaluated the model's performance on normal cancer and TNBC. As is shown in Figure 3, when the false‐positive level is 0.25, the model's sensitivity for TNBC (0.75) is lower than the average level (0.85) and *p*‐value is 0.073.

**FIGURE 4:**
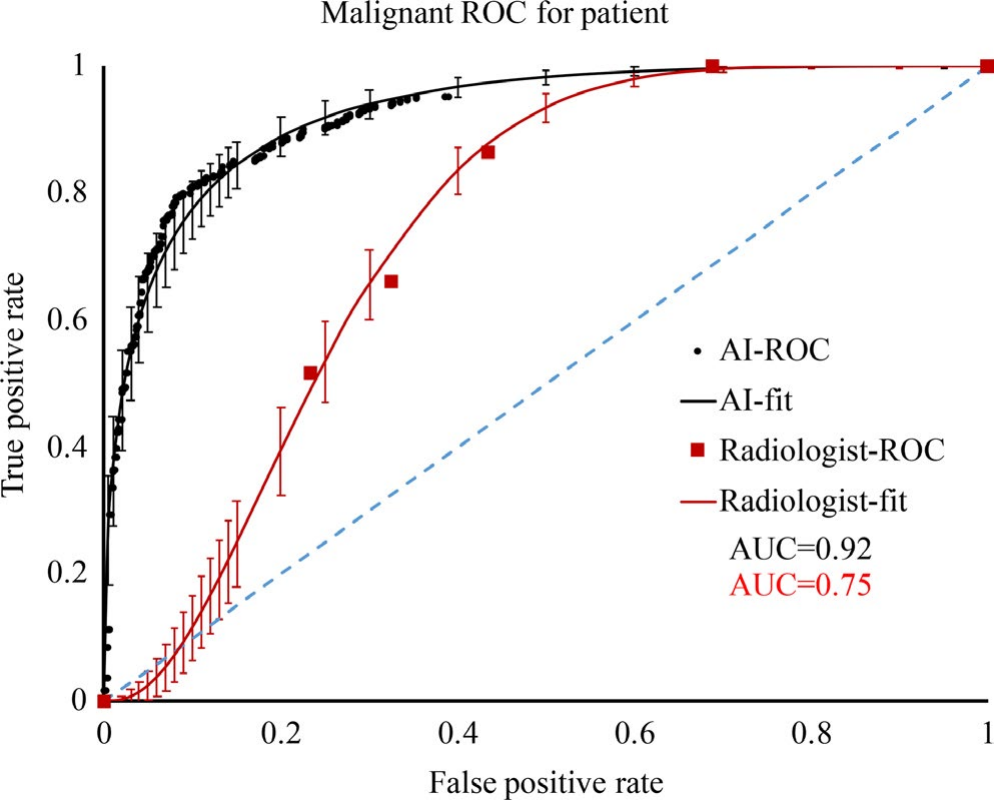
Receiver operating characteristic (ROC) curve on patient level. Set the max value of malignancy of all lesions as the malignancy value of the patient's level. Then, the ROC curve (black solid circle) was plotted from the breast level malignancy value and area under the curve (AUC) = 0.92. We also plotted the ROC curve (red square) using the six points Breast Imaging Reporting and Data System scale method. The solid lines are the ROC curve fitting curve, and the 95% CI is represented by the vertical bars. The dash blue line's AUC = 0.5 indicates that the model is completely meaningless

The third module malignancy prediction network in breast Yizhun AI system made a malignancy prediction for each lesion followed by the lesion matching module. Malignancy score is a continues real number value range is [0,1]. We chose the max malignancy score on all of the lesions of the two views on one side breast as the breast malignancy and plotted a ROC curve on the patient's level (Figure 4). The area under the curve (AUC) = 0.92 and the 95% CI was (0.902–0.936) which is obtained by the bootstrap method. Compared to the results of Kim et al.,^22^ the performance of our model has achieved state‐of‐the‐art classification accuracy on the malignancy prediction task. For the human reader, we adopted the six‐point BI‐RADS scale method, which has been reported in previous work,^11^ to evaluate the malignancy prediction performance of radiologists. The AUC of the radiologist was 0.75, which was similar to the results mentioned in a previous study.^8^, ^11^ While the BI‐RADS scale is not an optimal tool for ROC analysis,^22^ because its assessment categories do not constitute and ordinal scale. The seven‐point scale reported on the reference^8^ is a more suitable way for ROC analysis of lesions' malignancy. There are 33 sites' radiologists received the seven‐points malignancy scale training and they found that the radiologist's AUC can reach 0.84 in women under the age of 50 years.

Clinically, we prefer the model output a negative or positive prediction, so we find the optimal cut‐point by using the concordance probability method.^23^ In our model, the sensitivity and specificity at the optimal cut‐point are 0.844 and 0.866, respectively. Kappa value between model and golden standard was 0.693 (CI: 0.649, 0.735) and approximate significance <0.001.

## 4 DISCUSSION

The edge, shape, density, and microcalcification of the breast mass are vital signs in breast cancer diagnosis by mammography. There are a variety of single or combined deep learning models for these signs, which mainly focus on the classification of breast masses, detection of calcification foci, and early risk prediction in breast cancer. Kooi et al.^24^ developed a model to distinguish benign isolated cysts from malignant masses, using tissue enhancement to stabilize the overlapping tissues, with an accuracy of up to 80%. In order to screen the classification model of microcalcification, Wang et al.^25^ explored the influence of different convolutional layer structures on the classification performance. The study showed that increasing the number of filters in the convolutional layer can significantly improve microcalcification classification accuracy. Sun et al.^26^ developed and test a new near‐term breast cancer risk prediction scheme based on the quantitative analysis of the ipsilateral view of the negative screening mammograms. The results showed that the AUC of this model for breast cancer diagnosis is 0.737 ± 0.052, indicating that deep learning has great potential in developing a risk prediction model for early breast cancer. However, there are only a few studies on the detection of breast lesions in different density types (A, B, C, D). The difference in breast density exerts a significant influence on the detection and diagnosis of breast lesions, and this objective factor cannot be ignored. Therefore, the current study provides important information in this field and has great reference value.

In this study, we have shown that the Yizhun Medical Yizhun AI’s mammography auto diagnostic system reached state of the art about detection and diagnostic performance. The detection rate about calcifications has reached 90% at the false‐positive level of 0.25 per image in the validation data set, and the detection rate of the calcification lesions on breasts B, C, and D are almost similar. In the occlusion of breast glandular nodes, the mass, distortion, or asymmetrical lesions are not detected easily on the D‐type breast. Our model could find 72% of the three types of lesions when there are 0.25 false‐positive lesions per image, and for other types of breast, the sensitivity has reached 0.9. The artificial intelligence based computer‐aided detection (Yizhun AI‐CAD) system mentioned described by Kooi et al.^24^ showed that the sensitivity of lesion detection reached 0.7 when the false‐positive rate is 0.3.^27^ The study compared the Yizhun AI‐CAD results with three experienced radiologists; of whom two showed similar performance with Yizhun AI‐CAD. The advantage of our model is due to the following two reasons: a two‐stage detection network Mask‐rcnn which shows the best performance on object detection, and the other is a lesion matching module, designed for mammography.

The performance of the Yizhun AI system on TNBC was not as good as the average performance. We made a Chi‐square test *p* = 0.073 to evaluate the difference of model's performance on overall cancer and TNBC and *p* = 0.053 for the non‐TNBC and TNBC. TNBC lesions were commonly presented mammographically as irregular non‐calcified mass with ill‐defined or spiculated margins^28^ which are also difficult for medical imaging diagnostic.

To compare the malignancy prediction of the model with other studies,^11^, ^22^ we plotted the ROC curve. In our model, the AUC reached 0.92 and this value in Kim's study was 0.959. This could be attributed due to the smaller training data set scale of our model (58516 cases) than the reference (170,230 cases from three countries).

The first CAD system for clinical use in screening mammography was approved by the US FDA in 1988. Traditional CAD cannot improve the screening performance significantly, which has been proved by one of the largest studies.^16^ Also, some evidence has shown that its use led to high false‐positive rates, sensitivity rates, and biopsy rates, while the sensitivity of the cancer detection rates is not increased.^29^ Traditional CAD algorithms consist of mathematical models that depend on the handcrafted features, which require consultation with radiologists during development and are thus biased toward human thought processes.^29^ In the current study, our results outperform the deep learning model developed in 2017,^27^ indicating that the deep learning model is better than the traditional CAD model. In the next study, we will carry out a large‐scale multicenter prospective reader study to evaluate the clinical performance of the model.

Although deep learning has developed rapidly in the field of mammography, it still faces great challenges. First, it is extremely difficult to establish a unified mammography database because it requires multiple units to cooperate in formulating unified standards. Second, the large number of neural network parameters affects the operation speed. Thus, the network structure should be improved continuously to raise the computing speed on the premise of ensuring the accuracy of diagnosis. Third, the sustainable development of the deep learning model in the future cannot be achieved without the cooperation of imaging doctors and computer experts. Therefore, it is necessary to optimize the model structure continually. Despite several difficulties, the rapid development of deep learning has continuously expanded the boundary of medical imaging in recent years. In the future, this tool will play a great role in the diagnosis of breast diseases and prove to be a valuable assistant for radiologists.

## CONFLICT OF INTEREST

The authors declare that they have no competing interests.

## AUTHOR CONTRIBUTION

Hongmei Li and Jing Ye conceptualized the study, analyzed the mammography data in this study, and drafted the article. Hao Liu and Yichuan Wang released the annotation tool for physicians' annotation task. Hongmei Li and Hao Liu designed the experiments. Hongmei Li and Hao Liu realized the experiment in the part of the classification module. Binbin Shi helped to check the grammar of this article. Juan Chen and Ai ping Kong collected the data and organized the annotation task. Qing Xu and Jun hui Cai revised the article. All authors read and approved the final manuscript.

## ETHICS APPROVAL

This study was approved by the Medical Ethics Committee of Subei People's Hospital of Jiangsu Province.

## Data Availability

The data set supporting the results of this article is included within the article. The data sets used and/or analyzed during the current study are available from the corresponding author on reasonable request.
